# The impact of awareness of health checkup results on dropout from the specific health guidance programs on metabolic syndrome in the teacher population

**DOI:** 10.1265/ehpm.25-00365

**Published:** 2026-02-26

**Authors:** Kyoko Nakao, Yoshino Yokoyama, Hiroo Ide, Kazuhiko Kotani, Yuji Furui

**Affiliations:** 1 Healthcare Data Science Research Unit, Institute for Future Initiatives, The University of Tokyo, Bunkyo-Ku, Tokyo, Japan; 2 Division of Community and Family Medicine, Center for Community Medicine, Jichi Medical University, Shimotsuke-City, Tochigi, Japan; 3 Graduate School of Health Data Science, Juntendo University, Urayasu, Chiba, Japan

**Keywords:** Awareness, Health checkup, Health administration, Metabolic syndrome, Dropout

## Abstract

**Background:**

In Japan, specific health guidance is a program based on health checkups to prevent metabolic syndrome. However, dropout from the program is an issue and reduces opportunities to improve cardiovascular risk. Lack of awareness of health checkup results is thought to be associated with dropout, but the association remains unclear. This study aimed to investigate the impact of awareness of health checkup results with related attitude on dropout from specific health guidance among teachers.

**Methods:**

Data from medical insurance providers primarily serving teachers (n = 7,031; mean age = 51.19 years old) were analysed. Participants were categorized into 3 groups based on awareness of health checkup results and attitude toward adopting healthy lifestyles at the first time of the health guidance: awareness with the attitude (level 1 group), awareness without the attitude (level 2 group), and lack of awareness (level 3 group). Dropout rates from specific health guidance and improvement in health checkup results in the following year were compared across groups.

**Results:**

Dropout rates were 19.88% in the level 1 group, 22.03% in the level 2 group, and 27.77% in the level 3 group (*P* < 0.001 in a trend test). Compared with the level 1 group (reference), adjusted risk ratios for dropout were 1.139 (95% confidence interval: 1.001–1.295) for the level 2 group and 1.320 (95% confidence interval: 1.132–1.540) for the level 3 group. The degree of improvement in health checkup results decreased progressively from level 1 to level 3.

**Conclusions:**

Among the participants who received the first health guidance, awareness of health checkup results combined with attitude to adopt healthy lifestyles was associated with lower dropout from specific health guidance and greater improvements in cardiovascular risk. Such individuals’ awareness and attitudes may help predict individuals at risk of dropout from health guidance in this population.

## 1. Introduction

Metabolic syndrome, a lifestyle-related disorder characterized by obesity and insulin resistance, increases the risk of cardiovascular disease and death [1, 2]. Its increasing prevalence in the working population contributes to long-term absenteeism, posing challenges for workforce stability [3]. In 2008, Japan introduced a nationwide screening system called “specific health checkups” and a nationwide lifestyle intervention called “specific health guidance.” Following screening, medical insurers provide the health guidance to enrollees aged 40 to 74 years who are at risk of metabolic syndrome [4]. Specifically, the guidance is provided to participants with excessive waist circumference (WC) or body mass index (BMI) and at least 2 additional criteria, including elevated blood pressure, blood sugar, or lipid levels, or current smoking [4]. Health professionals such as public health nurses and dietitians provide the guidance for at least 3 months [4]. Participation in specific health guidance has been shown to promote weight loss and reduce the risk of lifestyle-related diseases [5–7].

Despite the benefits of the program, several challenges remain. For instance, not all eligible individuals receive the guidance after the health checkups [8], and some participants drop out of the program before completion [9]. Because dropout from medical interventions is associated with poorer health outcomes [10], reducing dropout and promoting health behaviours are important for achieving the goals of specific health guidance [11]. Despite this, no studies have yet examined dropout in the context of specific health guidance.

Previous research suggests that access to and understanding of health information can promote acceptance of health services and behaviours [12–14]. Participants’ awareness of their health risks and their attitude toward risk modification may therefore affect acceptance of specific health guidance. In addition, in some cases, inconsistencies have been observed between awareness of health checkup results and the attitude to adopt healthy lifestyles. An earlier study showed that accessing health information and taking action on it are components of the knowledge-attitude-practice model of health behaviour [15]. In this context, dropout should be studied with consideration of both awareness of checkup results and attitude to adapt healthy lifestyles.

As a working population, teachers have been studied to improve metabolic risk [16–18]. To control metabolic syndrome among teachers, their awareness and related attitude towards their health conditions would be explored. This study aimed to investigate the impact of awareness of health checkup results and attitude towards adopting healthy lifestyles on dropout from specific health guidance among teachers.

## 2. Methods

### 2.1 Participants

We analysed data from medical insurance providers primarily serving teachers. Participants were all teachers enrolled in medical insurance programs who received specific health checkups and specific health guidance in 2020 (n = 7,031). Participants met the criteria for support in specific health guidance; in this case, those taking medications or medical treatments for lifestyle-related diseases were excluded in the rule of the guidance [4]. This study was approved by the ethical review board of the University of Tokyo (the University of Tokyo, no. 23-336).

### 2.2 Measures

Each participant received the results of their health checkup by sealed letter about 1 month after the checkup, and specific health guidance started about 2 months later, which was a regular course. Participants’ awareness of their health checkup results was assessed by medical professionals such as public health nurses and dietitians at the first time of the health guidance. Awareness was determined by asking whether participants confirmed and remembered their health checkup results and whether they had attitude to adopt health behaviours such as making lifestyle changes after receiving their results. The determination of awareness was performed through the uniformed way of interview. Participants did not receive a direct explanation of their health checkup results at the first time of the health guidance.

Participants were categorized into 3 groups based on awareness and attitude. The “level 1” group included participants who were aware of their results and showed attitude to adopt healthy lifestyles. The “level 2” group included those who were aware of their results but did not show attitude to adopt healthy lifestyles. The “level 3” group included those who either did not remember their results or had not reviewed them. Thus, the level 1 group was considered to have the highest awareness (as a reference), while the level 3 group had the lowest.

The primary outcome was the rate of dropout from specific health guidance, defined as discontinuation of guidance or failure to complete the final evaluation. Completed was defined as receiving at least 3 months of guidance. The secondary outcome was the degree of improvement in health checkup results in the following year of the guidance.

Health checkup values, including body composition and blood biochemical tests, were measured with standardized methods for Japanese specific health checkups [4]. Improvement in results was evaluated based on reductions in BMI, WC, systolic blood pressure (SBP), diastolic blood pressure (DBP), triglycerides (TG), low-density lipoprotein cholesterol (LDL-chol), and hemoglobin A1c (HbA1c), and increases in high-density lipoprotein cholesterol (HDL-chol).

### 2.3 Analytic strategy

Participants’ characteristics, health status, and lifestyle habits were compared among the three awareness groups. Modified Poisson regression analysis [19, 20] with robust error variance was used to estimate risk ratio (RR) for dropout risk. In this study, adjusted RR (aRR) was further calculated after adjusting for covariates that were age, gender, and health checkup results at the first time of the guidance. Improvement in health checkup results the following year were compared among groups using analysis of covariance. Because poorer baseline may allow greater improvement partly due to regression to the mean [21], health checkup results at the first time of the guidance were included as covariates, along with age and gender, which are assumed to affect the amount of change in results [22–24]. Tukey’s multiple comparison test was used to identify significant differences among the groups. The analysis was conducted using R version 4.4.2, and the two-sided significance level was set at .05.

## 3. Results

### 3.1 Participant characteristics

The characteristics, health checkup results, and lifestyle habits of participants differed across groups (Table 1). Significant differences across groups were found in BMI, WC, SBP, DBP, TG, LDL-chol, and HbA1c. The values of BMI, WC, SBP, DBP, TG, and LDL-chol appeared highest in the level 3 group, with a tendency for higher values as awareness levels were lower. Across groups, significant differences were found in the proportion of participants who smoked, exercised, and ate dinner late. The proportion of participants who smoked and ate dinner late appeared highest in the level 3 group.

**Table 1:** Characteristics of the participants by the level of awareness of health checkup results

**Characteristic**	**All** ***(n = 7,031)***	**Level 1** ^a^ ***(n = 1,504)***	**Level 2** ^b^ ***(n = 4,317)***	**Level 3** ^c^ ***(n = 1,210)***	***P*-value** ^d^
Male, n (%)	5,957 (84.72%)	1,254 (83.38%)	3,657 (84.71%)	1,046 (86.45%)	0.087
Age (years)	51.19 (6.31)	51.36 (6.27)	51.13 (6.28)	51.21 (6.47)	0.500
Body mass index (kg/m^2^)	27.54 (3.51)	27.42 (3.45)	27.47 (3.41)	27.94 (3.86)	<0.001
Waist circumference (cm)	93.98 (7.73)	93.60 (7.54)	93.88 (7.51)	94.83 (8.62)	<0.001
Systolic blood pressure (mmHg)	134.06 (15.81)	133.68 (14.52)	133.70 (15.66)	135.80 (17.65)	<0.001
Diastolic blood pressure (mmHg)	85.71 (11.24)	85.21 (10.55)	85.45 (11.12)	87.27 (12.32)	<0.001
Triglycerides (mg/dL)	161 (108, 215)	154 (103, 207)	162 (109, 215)	164 (115, 221)	<0.001
LDL cholesterol (mg/dL)	139.58 (32.11)	137.18 (30.93)	139.49 (31.97)	142.90 (33.75)	<0.001
HDL cholesterol (mg/dL)	52.03 (12.00)	52.64 (12.28)	51.91 (11.91)	51.70 (11.97)	0.082
HbA1c (%)	5.82 (0.74)	5.82 (0.75)	5.79 (0.66)	5.93 (0.96)	<0.001
Smoking, n (%)	2,240 (31.86%)	437 (29.06%)	1,359 (31.48%)	444 (36.69%)	<0.001
Exercise,^e^ n (%)	1,722 (24.49%)	388 (25.80%)	1,069 (24.76%)	265 (21.90%)	0.012
Daily physical activity,^f^ n (%)	3,324 (47.28%)	685 (45.55%)	2,081 (48.20%)	558 (46.12%)	0.300
Skipping breakfast,^g^ n (%)	2,722 (38.71%)	549 (36.50%)	1,670 (38.68%)	503 (41.57%)	0.140
Eating dinner late,^h^ n (%)	959 (13.64%)	176 (11.70%)	558 (12.93%)	225 (18.60%)	<0.001
Drinking alcohol, n (%)					0.400
Every day	2,115 (30.08%)	460 (30.59%)	1,291 (29.91%)	364 (30.08%)	
Sometimes	2,305 (32.78%)	520 (34.57%)	1,391 (32.22%)	394 (32.56%)	
Never	2,470 (35.13%)	498 (33.11%)	1,545 (35.79%)	427 (35.29%)	
Sufficient rest from sleep, n (%)	4,265 (60.66%)	909 (60.44%)	2,632 (60.97%)	724 (59.83%)	0.200
Medical history,^i^ n (%)	150 (2.13%)	47 (3.13%)	88 (2.04%)	15 (1.24%)	0.003

Values represent means (standard deviations), unless otherwise indicated. Triglycerides represent the median (interquartile range).

Abbreviations: LDL, low-density lipoprotein; HDL, high-density lipoprotein; HbA1c, hemoglobin A1c.

^a^Level 1, aware with attitude group.

^b^Level 2, aware without attitude group.

^c^Level 3, unaware group.

^d^Pearson’s Chi-squared test; one-way ANOVA; Triglyceride values were log-transformed.

^e^Exercise for at least half an hour at least twice per week.

^f^Walking or equivalent physical activity for at least 1 hour per day.

^g^Skipping breakfast at least 3 times per week.

^h^Eating dinner within 2 hours before going to bed at least 3 times per week.

^i^Medical history shows the past history of chronic kidney disease, heart disease, or stroke.

### 3.2 Risk of dropout

Dropout rates were 19.88% in the level 1 group, 22.03% in the level 2 group, and 27.77% in the level 3 group. Compared with the level 1 group, the level 2 group’s crude RR for dropout was 1.108 (95% confidence interval [CI, 0.987–1.244]), and the level 3 group’s crude RR was 1.397 (95% CI [1.219–1.601]) (Table 2). After adjustment for covariates, the level 2 group had an aRR of 1.139 (95% CI [1.001–1.295]), and the level 3 group had an aRR of 1.320 (95% CI [1.132–1.540]).

**Table 2:** Dropout risk ratios

		**Univariate analysis**			**Multivariate analysis**		
		**RR**	**(95% CI)**	***P*-value**	**aRR**	**(95% CI)**	***P*-value**
Gender	Male	-			Reference		
	Female	-			1.108	(0.961–1.278)	0.158
Age (years)		-			1.000	(0.993–1.008)	0.926
Body mass index (kg/m^2^)		-			0.995	(0.968–1.023)	0.734
Waist circumference (cm)		-			1.009	(0.997–1.021)	0.135
Systolic blood pressure (mmHg)		-			1.002	(0.999–1.005)	0.190
Triglycerides (mg/dL)		-			1.001	(1.000–1.001)	0.001
LDL cholesterol (mg/dL)		-			1.001	(1.000–1.003)	0.099
HbA1c (%)		-			1.037	(0.978–1.099)	0.225
Awareness category	Level 1^a^	Reference			Reference		
	Level 2^b^	1.108	(0.987–1.244)	0.083	1.139	(1.001–1.295)	0.048
	Level 3^c^	1.397	(1.219–1.601)	<0.001	1.320	(1.132–1.540)	<0.001

Abbreviations: RR, risk ratio; CI, confidence interval; aRR, adjusted risk ratio; LDL, low-density lipoprotein; HbA1c, hemoglobin A1c.

^a^Level 1, aware with attitude group.

^b^Level 2, aware without attitude group.

^c^Level 3, unaware group.

### 3.3 Improvement in health checkup results after guidance

**Fig. 1:**
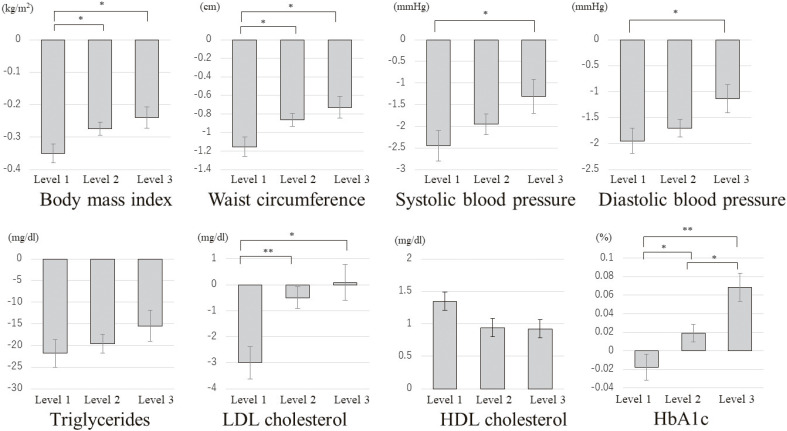
Estimated average change in health checkup results in the following year Note: The extent of improvement in test values was estimated by adjusting for gender, age, and pre-guidance test values. * P < 0.05, ** P < 0.01. The bar is standard error range. Abbreviations; LDL, low-density lipoprotein; HDL, high-density lipoprotein; HbA1c, hemoglobin A1c.

Adjusted analysis of covariance showed that improvement in health checkup results in the following year was greatest in the level 1 group, followed by the level 2 and 3 groups (Fig. 1). The values for BMI, WC, HbA1c, and LDL-chol differed significantly between the level 1 vs. 2 and level 1 vs. 3 groups. Additionally, SBP and DBP values differed significantly between the level 1 and 3 groups. TG and HDL-chol values showed a similar decreasing trend across groups, although the differences were not statistically significant.

## 4. Discussion

To our knowledge, this is the first study to provide basic data on the impact of awareness of health checkup results on dropout from specific health guidance. In this teacher population, participants who lacked awareness of their health checkup results were more likely to drop out from health guidance than those who were aware of their results and showed attitude to adopt healthy lifestyles. In addition, those without awareness of their results exhibited less improvement in their results after receiving the guidance. Because awareness of results and attitude to adopt healthy lifestyles can be easily assessed by interview, it could help predict individuals at risk of dropout from health guidance.

The association between awareness of health checkup results, attitude, and dropout from health guidance is reasonable. Health checkup results partly reflect a participant’s health status, and greater awareness may heighten perceived health risks, motivating adherence to guidance. Likewise, a high level of attitude to adopt healthy lifestyles may promote adoption of the behaviours recommended by guidance. Even though the participants’ occupations and the method to return their health checkup results (i.e., by letter) were the same, their awareness and attitudes were heterogenous. This may reflect individual differences in medical history or interest in health. The method by which health checkup results are returned to participants may also be considered as a factor for increasing awareness. In many cases, health checkup results are simply mailed, which may not sufficiently engage recipients. Further research on effective methods to present checkup results [25] and offer personalised, easy-to-navigate health information [26] are necessary.

Participants with high awareness and high attitude showed the greatest improvement in health checkup results, suggesting that they made lifestyle changes or received medical care after the guidance. This is consistent with the consensus on the favourable effect of guidance-oriented practice on cardiovascular risk [27]. Specifically, the level 1 group showed greater improvements in BMI, WC, LDL-chol, and HbA1c than the level 2 group. Although both groups were aware of their health checkup results, the level 1 group had higher attitude to adopt healthy lifestyles, suggesting that importance of the attitude is important for improving cardiovascular risk. While most health checkup results tended to improve in the following year across 3 groups, HbA1c values improved significantly in the level 1, but not the level 2 and 3 group. This reason for this finding is unclear but may relate to the unique stress profile of teachers, as mental stress can affect glucose metabolism [18]. Additional research is needed to clarify this result.

This study has several strengths and limitations. A strength is that awareness was assessed from the questions through the uniformed way of interviews by medical professionals rather than participant self-reports, reducing potential social desirability bias [28]. While the guidance was designed to follow standardized Japanese health checkup protocols [4], the specific content of the guidance was not evaluated. If guidance was intensified for participants with low awareness, the group differences may have been underestimated. Although the participants who received the first health guidance were analysed, we did not see the data of those who did not participate in the guidance. Awareness, attitude, and lifestyle are generally composed of complex factors; possibly, there remains to be unexamined confounding factors (e.g., health literacy and mental stress [18]) for analysis. Awareness was applied to the study as it was assessed in the real settings of guidance by a simple interview; however, considering that awareness is associated with health literacy, we must incorporate validated measures such as the European Health Literacy Survey Questionnaire [29] or the Health Literacy Questionnaire [30]. The participants’ awareness and attitude were assessed only at the first time of the guidance, and changes in awareness and attitude during the guidance were not examined. Addressing these limitations in future research will provide clearer understanding.

## 5. Conclusion

In this study of teachers, those who were not aware of their health checkup results had a higher risk of dropping out from health guidance than those who were aware of their results and showed attitude to healthy lifestyles. In addition, those who were not aware of their results showed less improvement in their results after the guidance. These findings indicate that awareness of health checkup results and attitude to healthy lifestyles at the first health guidance could predict individuals at the risk of dropout from the guidance and poor health improvement.

## Abbreviations

WC: waist circumference
BMI: body mass index
SBP: systolic blood pressure
DBP: diastolic blood pressure
TG: triglycerides
LDL-chol: low-density lipoprotein cholesterol
HDL-chol: high-density lipoprotein cholesterol
HbA1c: hemoglobin A1c
RR: risk ratio
aRR: adjusted risk ratio

## Declarations

### Availability of data and materials

The source data is not openly available for privacy or ethical reasons.
